# Selective cytotoxicity of Pancratistatin-related natural *Amaryllidaceae *alkaloids: evaluation of the activity of two new compounds

**DOI:** 10.1186/1475-2867-7-10

**Published:** 2007-06-05

**Authors:** Carly Griffin, Natasha Sharda, Divya Sood, Jerald Nair, James McNulty, Siyaram Pandey

**Affiliations:** 1 Department of Chemistry and Biochemistry, University of Windsor, Windsor, Ontario, Canada; 2 Department of Chemistry, McMaster University, Hamilton, Ontario, Canada

## Abstract

**Background:**

Pancratistatin (PST), a compound extracted from an *Amaryllidaceae *(AMD) family plant, has been shown to specifically induce apoptosis in cancer cells with no/minimal toxic effect on normal cells. A systematic synthetic approach has indicated that the minimum cytotoxic pharmacophore comprises the *trans*-fused b/c-ring system containing the 2, 3, 4-triol unit in the C-ring. To further explore the structure-activity relationship of this group of compounds we have investigated the anti-cancer efficacy and specificity of two PST-related natural compounds, AMD4 and AMD5. Both of these compounds lack the polyhydroxylated lycorane element of PST instead having a methoxy-substuituted crinane skeleton.

**Results:**

Our results indicate that AMD5 has efficacy and selectivity similar to PST, albeit at a 10-fold increased concentration. Interestingly AMD4 lacks apoptotic activity.

**Conclusion:**

Our results indicate that the phenanthridone skeleton in natural *Amaryllidaceae *alkaloids may be a significant common element for selectivity against cancer cells; furthermore, the configuration of the methoxy-side groups is responsible for higher binding affinity to the target protein/s thus making for a more efficient anti-cancer agent.

## Background

The initial report by Pettit and colleagues in 1993 demonstrated that Pancratistatin (PST) a natural compound isolated from the Hawaiian spider lily (*Hymenocallis littoralis*) displayed potent cytotoxicity against human tumour cell lines [1]. They also noted that in the NCI "disease oriented" *in vitro *anti-tumour screen against 60 cell lines representing lung, colon, ovarian, renal, brain, melanoma and myelocytic leukemia, the pattern of PST's activity against the various tumour types was distinct from that of other known anti-tumour drug classes. Recently, we demonstrated the selective toxicity of PST to cancer cells and the sparing of normal cells at micromolar doses [2]. In addition we have demonstrated that PST acts by affecting mitochondrial function and inducing apoptosis in malignant cell lines [3].

A systematic synthetic approach has been used to determine the minimum cytotoxic pharmacophore and is known to comprise the *trans*-fused b/c-ring system containing the 2, 3, 4-triol unit in the C-ring [4-6]. It has also been noted that the 2, 3-diol derivatives have significant activity [7], indicating that the C3-hydroxyl is a moderating influence. The C7 phenolic and C1 hydroxyl functions are not essential [8,9].

**Figure 1:**
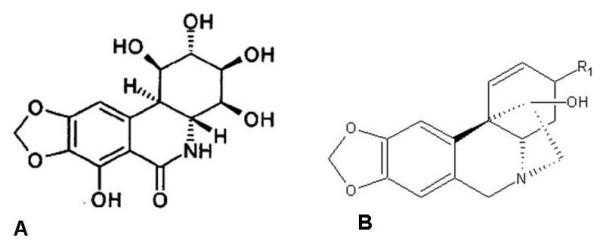
**Chemical structure of native Pancratistatin (A) and of Amaryllidaceae alkaloids AMD4 and AMD5 (B)**. The phenanthridone skeleton is a conserved feature in alkaloids of the *Amaryllidaceae *family. The Amaryllidaceae compounds have a methoxy group that varies in orientation between AMD4 (α) and AMD5 (β).

The aim of present work was to further evaluate the structure-activity relationships of two other AMD alkaloids, AMD4 and AMD5 (Figure 1). Both these compounds lack the multiple hydroxyl groups but instead have a methoxy group that varies in orientation between AMD4 (α) and AMD5 (β). Our results indicated that like PST, AMD5 has the capability of selectively inducing apoptosis in cancer cells while sparing normal cells, albeit at a concentration 10-fold higher.

## Results

### *Amaryllidaceae *alkaloids induce apoptosis in Jurkat cells

**Figure 2:**
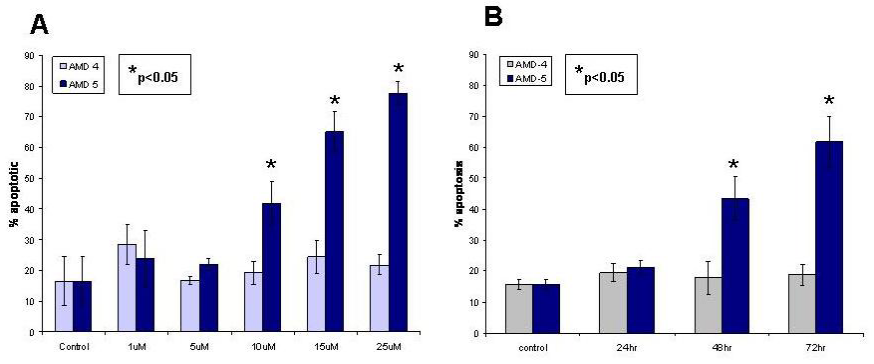
**A. Dose-response curve for Jurkat cells treated with various concentrations of either AMD4 or AMD5 for 48 hrs**. Cells were stained with cell-permeable Hoechst 33342 dye; brightly stained and condensed nuclei were considered to be apoptotic while non-bright, smooth nuclei were considered healthy (non-apoptotic). The degree of apoptosis was calculated by the number of apoptotic cells counted over the total number of cells visible and displayed as a percentage. A minimum of 5 fields with at least 100 cells per field were counted and tabulated using Microsoft Excel software; values that are statistically significant to p < 0.05 are indicated with an asterisk. Micromolar is represented as uM in this figure only. **B. Time Course: A measurement of the degree of apoptosis induced in Jurkat cells treated with 10 μM of either AMD4 or AMD5 over 72 hours**. Following treatment, Jurkat cells were incubated with cell-permeable Hoechst 33342 dye; brightly stained condensed nuclei were considered to be apoptotic while non-bright, smooth nuclei were considered healthy. The degree of apoptosis was calculated by the number of apoptotic cells counted over the total number of cells visible displayed as a percentage. A minimum of 5 fields with at least 100 cells per field were counted and tabulated using Microsoft Excel software; values that are statistically significant to p < 0.05 are indicated with an asterisk.

**Figure 3:**
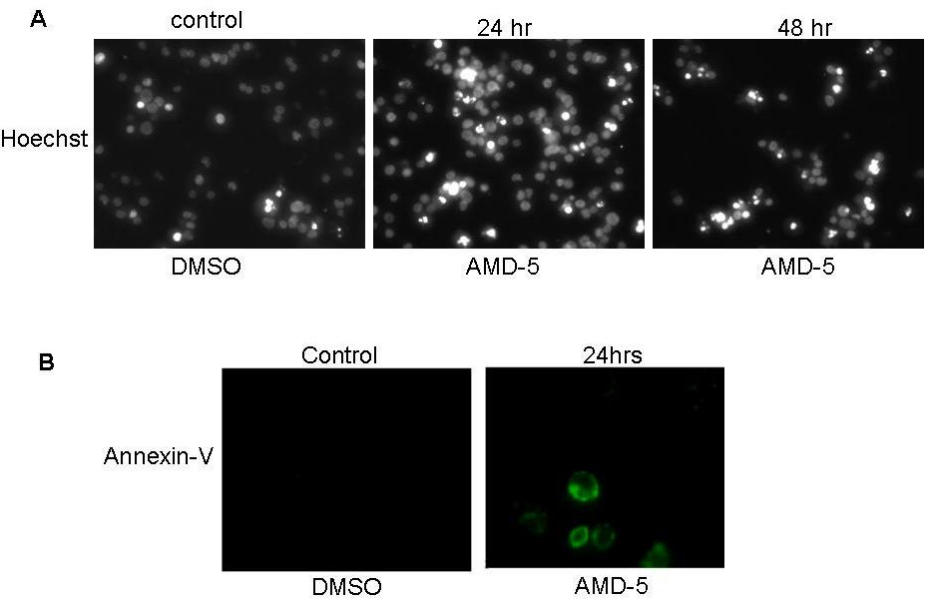
**A. Nuclear Morphology of Jurkat cells treated with AMD5 at 10 μM for either 24 hrs or 48 hrs**. Jurkat cells were stained with cell-permeable Hoechst 33342 dye to observe apoptotic nuclear morphology. Apoptotic nuclei are brightly stained and condensed when compared to healthy nuclei. The control was treated with DMSO solvent. Magnification: 200×. **B. Annexin-V Binding Assay: Jurkat cells were treated with 10 μM AMD5 for 24 hrs**. Jurkat cells were incubated with Annexin-V 488 Alexa-Fluor conjugate to observe phosphatidyl-serine flipping from the inner to the outer leaf of the plasma membrane. This is a characteristic event of apoptosis that is made visible with use of a fluorescent microscope; cells undergoing apoptosis will have more Annexin-V substrate bound, and thus appear brighter than healthy cells. Magnification: 400×

Jurkat cells were incubated with different concentrations of AMD4 and AMD5 for up to 72 hrs. The degree of apoptosis resulting from treatment was observed by Hoechst staining where condensed, brightly stained nuclei indicated apoptotic cell death. The number of apoptotic nuclei was expressed as a percentage of the total number of cells in a dose-dependent manner; alkaloid AMD5 at a concentration of 10 μM incited apoptosis in over 40% of Jurkat cells after 48 hrs and was the working concentration used for further experiments (Figures 2 &3A). In contrast to this finding, alkaloid AMD4 had a minimal effect on cancer cell viability under similar treatment conditions. To compliment Hoechst staining, the Annexin-V assay was carried out at several time-points in order to monitor phosphatidylserine flipping to the outer leaflet of the plasma membrane, a characteristic apoptotic event. As previously reported, Annexin-V staining is specific to apoptotic cells, and background staining is low in unaffected cells [11]. Jurkat cells incubated with 10 μM AMD5 resulted in a high incidence of phosphatidylserine flip; approximately 45% of Jurkat cells were observed to be labeled with Annexin-V-FITC (Figure 3B). Necrotic death in Jurkat cells after similar treatment times with either alkaloid was not observed by Trypan-blue staining, suggesting that cell death is apoptotic only (data not shown).

### DNA fragmentation observed in Jurkat cells treated with *Amaryllidaceae *alkaloids

**Figure 4:**
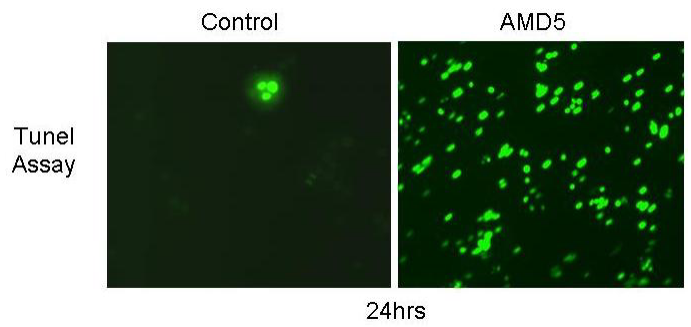
**TUNEL Assay: Jurkat cells were treated with AMD5 at 10 μM for 24 hrs**. Following treatment, Jurkat cells were fixed and immuno-stained with anti-BdrU antibody to observe the extent of DNA damage as described in the Materials and Methods section. The level of staining indicates the degree of DNA damage induced by treatment, where more positively stained cells are in the final stages of apoptosis. Magnification: 200×

The TUNEL (terminal transferase dUTP nick end labeling) assay was used to measure the extent of DNA fragmentation that occurs following treatment with alkaloid AMD5. Cells were grown and treated at 10 μM final concentration for 24 hrs. At this time-point, approximately 20% of cells displayed characteristic apoptotic morphology with Hoechst staining. As shown in figure 4, extensive DNA fragmentation was visible by fluorescence microscopy in cells treated with AMD5; this corresponds with the increase in apoptotic morphology at 48 hrs of treatment with this alkaloid.

### *Amaryllidaceae *alkaloid AMD5 causes early activation of caspase-3; disruption of the mitochondrial membrane potential delayed

**Figure 5:**
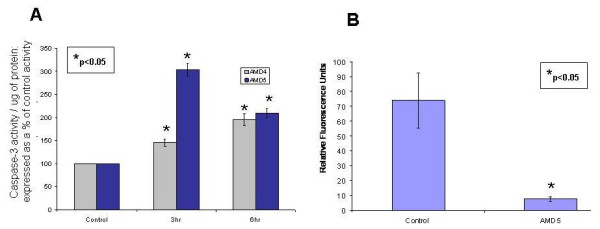
**A. Caspase-3 Assay: Jurkat cells were treated with 10 μM AMD4 or AMD5 for the early time-points of 3 hrs and 6 hrs**. Following treatment, Jurkat cells were incubated with DEVD-AFC peptide to detect caspase-3 activity; fluorescence was measured at excitation 400 nm and emission 505 nm. Activation of caspase-3 is a common event in cells undergoing apoptosis. The level of caspase-3 activity was calculated per microgram of protein and then displayed as a percent increase in fluorescence from caspase-3 activity in untreated cells (control). Values that are statistically significant to p < 0.05 are indicated with an asterisk. **B. Mito-Casp Assay: Jurkat cells were treated with 10 μM AMD5 for 48 hrs**. Following treatment, Jurkat cells were incubated with a mitochondrial membrane potential (MMP) sensitive dye, and fluorescence was measured at excitation 574 nm and emission at 595 nm. Loss of MMP indicates permeable mitochondria which allows for numerous cytotoxic materials to leak into the cytosol, ending in apoptosis. Loss of MMP is represented as a drop in relative fluorescence units per 10,000 cells. Values that are statistically significant to p < 0.05 are indicated with an asterisk.

The role of caspases as molecular mediators of apoptosis was examined after treatment up to 48 hrs with these alkaloids. A slight increase in caspase-3 activation was observed after 6 hrs in Jurkat cells treated with AMD4; however, a more drastic increase of 3 fold over control was incited after a 3 hr treatment with AMD5 (Figure 5A). At later treatment times of 24 and 48 hrs, caspase-3 activity reduced as found by the Mito-Casp assay (data not shown). This assay detects activation of caspases-3, -7 and -9 (executioner caspases) and also measures a change in mitochondrial membrane potential. Our results show that after 48 hrs the mitochondrial membrane potential has destabilized in correspondence with an increased number of apoptotic cells (Figure 5B). Destabilization of the mitochondrial membrane potential indicates leakage of cytotoxic mitochondrial contents into the cytoplasm, initiating the cell death process.

### Normal nucleated blood cells are unaffected by treatment with *Amaryllidaceae *alkaloid AMD5

**Figure 6:**
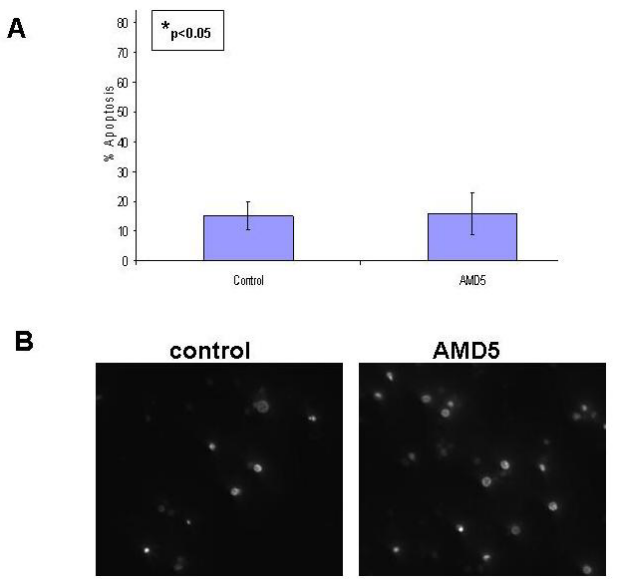
**A. Time Course: Degree of apoptosis in healthy peripheral mono-nucleated blood cells treated with 10 μM AMD5 for 48 hrs**. Healthy human peripheral mono-nucleated blood cells were collected & treated for 48 hours with 10 μM AMD5. The cells were then stained with cell-permeable Hoechst 33342 dye to observe apoptotic nuclear morphology. Brightly stained and condensed nuclei were considered apoptotic, non-bright smooth nuclei were considered healthy. The degree of apoptosis was calculated by the number of apoptotic cells counted over the total number of cells visible displayed as a percentage. A minimum of 5 fields with at least 100 cells per field were counted and tabulated using Microsoft Excel software; values that are statistically significant to p < 0.05 are indicated with an asterisk. **B. Nuclear Morphology: Healthy human peripheral mono-nucleated blood cells were collected & treated with 10 μM of AMD5 for 48 hrs**. Cells were stained with cell permeable Hoechst 33342 dye to observe apoptotic nuclear morphology. Apoptotic nuclei are brightly stained and condensed when compared to healthy nuclei. The control was treated with DMSO solvent. Magnification: 200×.

Mononuclear cells from peripheral venous blood were purified by centrifugation at 2900 rpm for 30 min at 37°C from whole blood obtained from a healthy volunteer. The cells were treated with 10 μM of AMD5 for 48 hrs, and then stained with Hoechst dye to observe apoptotic morphology. As shown in figure 6, this alkaloid had minimal effect on the viability of normal nucleated blood cells in comparison to untreated cells. This result suggests that AMD5 may be less toxic to healthy cells at a dosage capable of inducing apoptosis in approximately 45% of cancerous Jurkat cells after 48 hrs. As the effect of AMD4 against Jurkat cells was minimal, the specificity of this alkaloid was not tested against normal nucleated blood cells for specificity.

## Discussion

Here we observe that AMD5, a natural alkaloid from the *Amaryllidaceae *family, is capable of inducing apoptosis in more than 40% of Jurkat cells at a 10 μM concentration after 48 hrs treatment. In addition, this alkaloid was observed to have low toxicity to healthy mono-nucleated blood cells after 48 hrs treatment. This result suggests that crinane-type *Amaryllidaceae *alkaloids may possess anticancer activity similar to the lycorane derivatives related to PST. PST has been shown to be a highly specific anti-cancer agent, able to induce apoptosis in over 90% of Jurkat cells in only 24 hrs at 1 μM final concentration [11,14,15]. It appears that a correctly functionalized phenanthridone core may thus be a common minimal structural requirement for these cell specific anti-cancer agents. Although AMD5 was found to be well tolerated in non-cancerous cell lines in a cell culture model, its effects on physiological parameters in animal models should be evaluated for pharmacokinetic studies to determine the adsorption, distribution, metabolism, excretion and toxic properties.

An early increase in caspase-3 activation upon treatment with AMD5 suggests that an extrinsic pathway of apoptosis may be stimulated by this alkaloid. Our study also found that mitochondrial membrane potential was disrupted after 48 hrs, when over 40% of cells were apoptotic. This indicates that the intrinsic pathway of apoptosis, by which cytochrome *c *release from the mitochondria leads to a cascade of events that activates caspase-3 downstream, may not become activated. In comparison with AMD5, AMD4 had minimal effect against cancerous cells, which may indicate that the position of the methoxy side-group is crucial. AMD5, with the β-methoxy configuration, may have a stronger interaction with a critical protein versus AMD4, whose functional group is in the alpha configuration. This difference may account for our finding that AMD5 is a more efficient inducer of apoptosis in Jurkat cells than AMD4. It must be noted that the addition of an oxymethyl group has been shown to alter the activity of natural alkaloids [12] for example, researchers have observed increased cytotoxic activity of 8-methoxycaffeine compared to the parent compound [13]. However, the impact of the configuration of methoxy group and its varied effect on the activity of these AMD alkaloids is a novel and interesting observation.

## Conclusion

A correctly functionalized phenanthridone skeleton present in natural *Amaryllidaceae *alkaloids may be a significant common element for selectivity against cancer cells, furthermore, the configuration of the methoxy-side groups is responsible for higher binding affinity to the target protein/s thus making for a more efficient anti-cancer agent.

## Methods

### Cell cultures

Human T-cell leukemia (Jurkat) cells were obtained from ATCC, Manassas, VA. These cells were maintained in RPMI 1640 medium supplemented with 10% fetal bovine serum (FBS) and 10 μM gentamycin (Life Technologies, Mississauga, ON) in an incubator set at 37°C, 5% CO_2 _and 95% humidity.

Human nucleated blood cells were isolated from whole blood taken from a healthy non-smoking male according to the University of Windsor REB #04-147. The whole blood sample was collected in a BD Vacutainer™ CPT (Cell Preparation Tube) obtained from Becton Dickinson, Franklin Lakes, NJ, and spun at 2900 rpm for 30 min at 25°C. The upper layer consisting of mononuclear cells, platelets and plasma was collected and maintained with RPMI 1640 media supplemented with 10% FBS and 10 μM gentamycin (Life Technologies) in the same incubator as the Jurkat cells.

### Cell treatment

Cells were grown and treated with either AMD4 or AMD5 alkaloids at various concentrations and time-points. AMD4 and AMD5 (99.5% pure) were derived as detailed by Pettit *et al.*, 1993 [1].

### Cellular viability assay

Cells were grown to 70% confluence and treated with AMD4 and AMD5 at a final concentration of 10 μM. The cells were then stained with the cell permeable dye Hoechst 33342 (Molecular Probes, Eugene, OR) at 10 μM final concentration and incubated for 10 min at 25°C. Brightly stained, condensed nuclei are characteristic features of apoptotic cells, as visualized with a fluorescent microscope (Leica DM IRB, Germany). Images were captured at 10× and 40× objectives; the percentage of apoptotic cells was calculated from the total number of cells with Microsoft^® ^Excel 6.0 software and pictures were compiled using Adobe^® ^Photoshop 7.0 software. A minimum of 5 fields of at least 100 cells per field was counted. Statistical significance was determined using STATISTICA^® ^software.

### Annexin-V binding assay

Jurkat cells were treated with 10 μM AMD4 and AMD5 for 24 hours. The Annexin-V binding assay was conducted according to the manufacturer's protocol using a kit purchased from Molecular Probes, Eugene, OR. Briefly, cells were washed with phosphate-buffered saline (PBS) and re-suspended in Annexin-V binding buffer (10 mM HEPES/NaOH pH 7.5, 140 mM NaCl, 2.5 mM CaCl_2_), containing Annexin-V Alexa Fluor^® ^488 conjugate (1:50) for 15 min at 25°C; pictures were taken with a fluorescent microscope (Leica DM IRB, Germany) at 40× objective and compiled using Adobe^® ^Photoshop 7.0 software.

### TUNEL assay

After treating Jurkat cells with AMD4 and AMD5 at indicated times the TUNEL Assay was performed as per manufacturer's protocol (Molecular Probes, Eugene, OR) and a previously published method [10], to detect DNA damage. Cells were fixed by suspending them in 70% (v/v) ethanol and stored at -20°C overnight. The sample was then incubated with DNA-labeling solution (10 μL reaction buffer, 0.75 μL TdT enzyme, 8 μL BrdUTP, 31.25 μL of dH2O) for 1 hr at 25°C. Each sample was then exposed to an antibody solution consisting of 5 μL Alexa Fluor^® ^488 labeled anti-BrdU antibody with 95 μL rinse solution and allowed to react for 20 min; pictures were taken at 20× objective using a fluorescent microscope (Leica DM IRB, Germany).

### Caspase-3 assay

The caspase-3 assay was carried out using a previously published method [11]. Briefly, Jurkat or normal lymphocyte cellular lysates were collected and incubated with the fluorogenic substrate DEVD-AFC (MP Biomedicals, Aurora, OH) in DEVD buffer (0.1 M HEPES, pH 7.4, 2 mM DTT, 0.1% CHAPS, 1% sucrose) and allowed to incubate at 37°C for 45 min. Fluorescence was measured at 400 nm excitation and 505 nm emission using the Spectra Max Gemini XS (Molecular Devices, Sunnyvale, CA). Caspase-3 activity was calculated per microgram of protein, and expressed as a percentage of control activity. Protein concentration was determined utilizing the BioRad protein assay reagent (BioRad, Mississauga, ON, Canada) with bovine serum albumin as a standard. Microsoft^® ^Excel 6.0 software was used for data representation; statistical significance was determined using STATISTICA^® ^software.

### Mito-Casp assay

The Mito-Casp assay was performed as per manufacturer's protocol using a kit purchased from Cell Technology Inc., Mountain View, CA. Briefly, cells were treated with either alkaloid at 10 μM concentration for the desired times and washed twice in PBS. Cells were re-suspended in PBS according to protocol and incubated with both MMP dye and caspase reagent at a 1:30 dilution for 60 min at 37°C in darkness. Following incubation, the cell pellet was collected and re-suspended in wash buffer; fluorescence was measured from a 96-well micro-titre plate at 549 nm excitation and 574 nm emission using the Spectra Max Gemini XS (Molecular Devices, Sunnyvale, CA). Loss of MMP was presented as a loss in relative fluorescence units per 10,000 cells. Microsoft^® ^Excel 6.0 software was used for data representation; statistical significance was determined using STATISTICA^® ^software.

## Competing interests

The author(s) declare that they have no competing interests.

## Authors' contributions

CG carried out the viability and Annexin-V staining assays and drafted the manuscript. NS carried out the TUNEL assay. DS carried out the caspase-3 and mito-casp assays. JM and JJN provided the *Amaryllidaceae *alkaloids. SP conceived of the study, and participated in its design and coordination and helped to draft the manuscript. All authors read and approved the final manuscript.
